# Malaria Parasite-Infected Erythrocytes Secrete PfCK1, the *Plasmodium* Homologue of the Pleiotropic Protein Kinase Casein Kinase 1

**DOI:** 10.1371/journal.pone.0139591

**Published:** 2015-12-02

**Authors:** Dominique Dorin-Semblat, Claudia Demarta-Gatsi, Romain Hamelin, Florence Armand, Teresa Gil Carvalho, Marc Moniatte, Christian Doerig

**Affiliations:** 1 UMR S1134, Institut National de Transfusion Sanguine, 6 Rue Alexandre Cabanel, 75015 Paris, France; 2 Institut Pasteur, Unité de Biologie des Interactions Hôte-Parasites, 25–28 rue du Dr Roux, Paris F-75015, France; 3 Proteomics Core Facility, Ecole Polytechnique Fédérale de Lausanne, CH-1015 Lausanne, Switzerland; 4 Infection and Immunity Program, Monash Biomedicine Discovery Institute and Department of Microbiology, Monash University, Victoria, Australia, 3800; Université Pierre et Marie Curie, FRANCE

## Abstract

Casein kinase 1 (CK1) is a pleiotropic protein kinase implicated in several fundamental processes of eukaryotic cell biology. *Plasmodium falciparum* encodes a single CK1 isoform, PfCK1, that is expressed at all stages of the parasite’s life cycle. We have previously shown that the *pfck1* gene cannot be disrupted, but that the locus can be modified if no loss-of-function is incurred, suggesting an important role for this kinase in intra-erythrocytic asexual proliferation. Here, we report on the use of parasite lines expressing GFP- or His-tagged PfCK1 from the endogenous locus to investigate (i) the dynamics of PfCK1 localisation during the asexual cycle in red blood cells, and (ii) potential interactors of PfCK1, so as to gain insight into the involvement of the enzyme in specific cellular processes. Immunofluorescence analysis reveals a dynamic localisation of PfCK1, with evidence for a pool of the enzyme being directed to the membrane of the host erythrocyte in the early stages of infection, followed by a predominantly intra-parasite localisation in trophozoites and schizonts and association with micronemes in merozoites. Furthermore, we present strong evidence that a pool of enzymatically active PfCK1 is secreted into the culture supernatant, demonstrating that PfCK1 is an ectokinase. Our interactome experiments and ensuing kinase assays using recombinant PfCK1 to phosphorylate putative interactors *in vitro* suggest an involvement of PfCK1 in many cellular processes such as mRNA splicing, protein trafficking, ribosomal, and host cell invasion.

## Introduction

Human malaria is caused by infection with protozoan parasites of the genus *Plasmodium*, and *P*. *falciparum*, the most virulent species, is responsible for the vast majority of lethal cases. 40% of the world’s population is at risk of contracting the disease, and 300 million clinical cases as well as 650,000 deaths are reported yearly, mostly among young children in sub-Saharan Africa. Recently, a global drop in mortality has been observed thanks to the introduction of artemisinin-based combination therapies, preventive drug treatment, and mosquito control strategies, including massive distribution of insecticide-treated bednets. However, the rapid emergence and spread of resistance against the available anti-malarial armamentarium (including artemisinin-derived compounds [1]) and the resistance of the vector to insecticides cause great concern and urgently call for the development of new control agents. Research in malaria parasite biology, and in particular on asexual blood stages (the multiplicative stage of the life cycle associated with clinical manifestations) is expected to identify novel targets and thus to lead to the development of new therapeutic strategies.

Post-translational modifications act as molecular switches in eukaryotic cells to regulate the activity of many proteins, including those with key functions in fundamental cellular processes such as gene expression, cell division, cell differentiation and metabolism. Reversible phosphorylation of proteins mediated by protein kinases and phosphatases is a major post-translational modification: about one-third of eukaryotic proteins are phosphoproteins, and in mammalian cells this is mediated by >500 protein kinases and >200 phosphatases. The *P*. *falciparum* kinome and phosphatome comprise ~ 85 and ~30 enzymes, respectively [2,3]. Together with the fact that gene expression in malaria parasites is to a large extent regulated by post-translational mechanisms, this suggests that protein phosphorylation is an important feature in these organisms [4]. This is corroborated by the large number of phosphoproteins detected by mass spectrometry analyses [5–8]. In view of the success of targeting protein kinases in cancer chemotherapy, illustrated by the approval of several kinase inhibitors as anti-cancer drugs over the recent years [9], the *Plasmodium* kinome has been highlighted as a potential target for antimalarials with novel modes of action [10,11].

While some protein kinases are exquisitely selective with respect to their substrates, such as the MEKs (MAP/ERK kinases) that transduce signals in MAP kinase pathways, and whose only known substrate is their cognate MAP kinase [12], others phosphorylate a very large number of proteins and as such play pleiotropic roles in cell homeostasis. The seven isoforms of casein kinase 1 (CK1) found in mammalian cells collectively phosphorylate many different substrates, including regulators of a wide range of process such as cell differentiation and proliferation, transmembrane transport and circadian rhythm (reviewed in [13]), and the picture is very similar for the 5 CK1 isoforms found in yeast [14]. In contrast, the *P*. *falciparum* kinome contains a single member of the CK1 group [2,15]. Purified recombinant PfCK1 displays properties characteristic of CK1 group members, such as susceptibility to selective inhibitors of mammalian CK1 and ability to phosphorylate a peptide that is highly specific to CK1 enzymes [16]. Although PfCK1 is known from reverse genetics experiments to be essential for completion of the asexual intra-erythrocytic cycle [6], its cellular function and its sub-cellular localisation remain uncharacterised. Here, we demonstrate that PfCK1 is expressed throughout blood stages and localises not only in the parasite itself, but is also exported to the host erythrocyte; a significant pool of PfCK1 associates with the red blood cell surface at early stages of infection, and is selectively secreted into the culture medium. Interactomics experiments indicate that PfCK1 is likely implicated in numerous pathways and cellular processes, including mRNA splicing, invasion and chromatin dynamics, in line with the pleiotropic nature of its orthologues in mammalian cells.

## Materials and Methods

### Molecular cloning of PfCK1 and site-directed mutagenesis

The 970-bp PfCK1 coding sequence was amplified by PCR with Phusion Polymerase from a cDNA library and cloned into the bacterial expression vector pGEX4T3 between the BamH1 and Not1 sites, using the following primers: forward, GGGGGATCCATGGAAATTAGAGTGGCA AATAAATATG and reverse: GGGGCGGCCGCTTAATTTTGCTTTACTCTTCCT TC (restriction sites underlined). The resulting construct was verified by DNA sequencing prior to expression in bacteria. An expression plasmid encoding the K38M kinase-dead mutant was obtained by site directed mutagenesis using the overlap extension PCR technique using the following primers containing the mutation: Forward, GAATTTGCTGTAATGTTAGAATCAACACG; Reverse: CGTGTTGATTCTAACATTACAGCAAATTC (mutated codon underlined). The plasmid was sequenced to verify that no additional mutations had been generated during the PCR.

### Bacterial expression and purification of recombinant fusions proteins

Expression of GST (Glutathione-S-transferase) was performed in BL21 cells in media supplemented with 100μg/ml Ampicillin for 3 hours at 37C. Expression of GST-CK1 was performed respectively in Rosetta cells in media supplemented with 100μg/ml Ampicillin and 34μg/ml Chloramphenicol overnight at 20°C. Expression of both proteins was induced with 0.2mM IPTG at OD 0.5. The purification protocol was described previously [17].

### Parasite culture

Asexual parasites of the *P*. *falciparum* 3D7 clone were grown as described [18] and used as recipients in all transfections experiments. Synchronization of parasites was carried out by sorbitol treatment [19]. Gametocytes induction and culture were performed as previously described [20].

### Plasmids for parasite transfection

#### pCAM-BSD-KOPfCK1

A 1079 bp DNA fragment (nucleotides 48 to 1127 of the *pfck1* ORF) was amplified by PCR from *P*. *falciparum* genomic DNA, using primers (forward:GGGGGATCCGAGTGGTTCCTTTGGTGATATATATG; reverse GGGGCGGCCGCGGTAACTTTTTTCCCTCGTCC containing *Bam*H1 or *Not*1 sites (underlined) and was inserted at these sites into the pCAM-BSD vector [21].

#### pCAM-BSD-CK1GFP

The 3’end of the CK1 coding region (695 bp, omitting the stop codon) was amplified by PCR from genomic DNA, using the following primers: forward, GGGCTGCAGCCATGGCAAGGTCTAAAGGC; reverse, GGGGGATCCATTTTGCTTTACTCTTCCTTCTTG, with Pst1 and BamH1 restriction sites, respectively (underlined), which allowed insertion of the amplified product into pCAM-BSD-GFP.

#### pCAM-BSD-CK1HIS

The 3’end of the CK1 coding region (695 bp, omitting the stop codon) was amplified by PCR from genomic DNA, using the following primers: forward, GGGCTG CAGCCATGGCAAGGTCTAAAGGC; reverse, GGGAAGCTTTTAATGATGATGATGATGATGATGATTTTGCTTTACTCTTCCTTC, with Pst1 and HindIII restriction sites, respectively (underlined), which allowed insertion of the PCR product into pCAM- BSD.

### Parasite transfection

To disrupt the *pfck1* locus, ring stage parasites were electroporated with 60μg of pCAMBSD-CK1 plasmid DNA as described previously [6]. Blasticidin was added to a final concentration of 2.5μg/ml 24 hours after transfection. Resistant parasites appeared four weeks post transfection. To generate parasites expressing His- or GFP-tagged PfCK1, transfection of pCAM-BSD-CK1His or pCAM-BSD-CK1 GFP were performed as described previously.

### Genotype characterization

**Fig 1:**
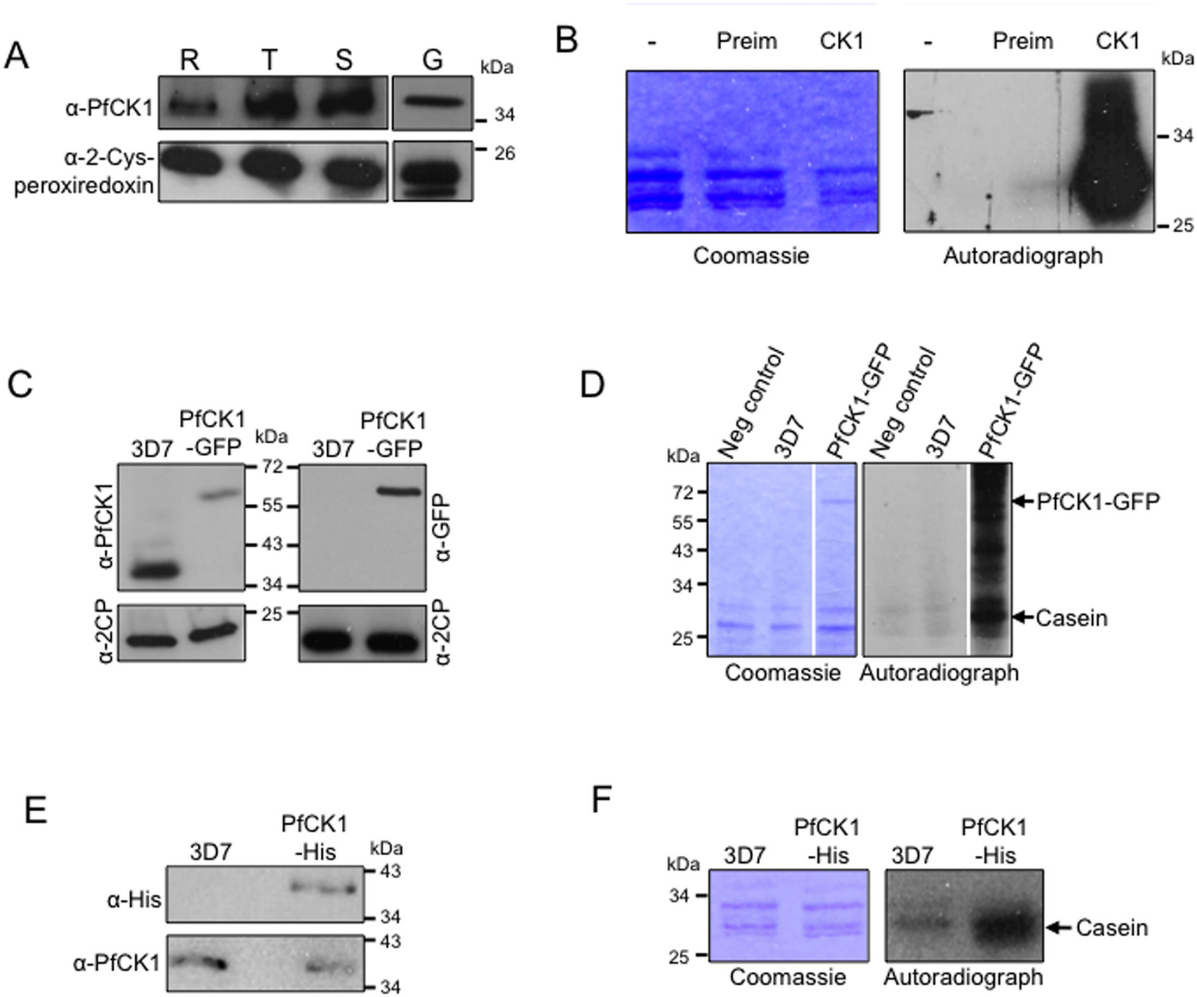
PfCK1 expression and kinase activity. (A) PfCK1 protein was detected by western blot using a peptide-derived anti-PfCK1 antibody. R: Rings; T: trophozoites; S: Schizonts; G: Gametocytes. Detection of 2-Cys-peroxiredoxin was used as a loading control (bottom panel). (B) Casein kinase activity immunoprecipitated with an anti-PfCK1 antibody from parasite extracts from a mixed asexual culture. Kinase assays were set up with casein as a substrate. Lane 1 (“-“), no immunoprecipitated added; lane 2 (“Preim”); immunoprecipitate obtained with a pre-immune serum lane 3 (“CK1”), immunoprecipitate obtained with the anti-PfCK1 antiserum. Reactions were subsequently analysed by SDS-PAGE and autoradiography. (C) Endogenously GFP tagged PfCK1. Western blot of parasite extracts from wild-type 3D7 parasites and parasites expressing GFP-tagged PfCK1 from the endogenous locus using anti-PfCK1, anti-GFP and anti-2-Cys-peroxiredoxin antibodies. (D) Kinase activity after immunoprecipitation from parasite extracts using an anti-GFP antibody. Kinase assays were set up with casein as a substrate. Lane 1, no immunoprecipitated added; lane 2, immunoprecipitate obtained from wild-type 3D7 parasites; lane 3, immunoprecipitate from parasites expressing GFP-tagged PfCK1 from the endogenous locus. Reactions were subsequently analysed by SDS-PAGE and autoradiography. (E) Endogenously His tagged PfCK1. Western Blot of parasite extracts from wild-type 3D7 parasites and parasites expressing His-tagged PfCK1 from the endogenous locus using anti-His and anti-PfCK1 antibodies. (F) Kinase activity after immunoprecipitation from parasite extracts using an anti-His antibody. Kinase assays were set up with casein as a substrate. Lane 1, immunoprecipitate obtained from wild-type 3D7 parasites; lane 2, immunoprecipitate from parasites expressing His-tagged PfCK1 from the endogenous locus. Reactions were subsequently analysed by SDS-PAGE and autoradiography.

For PCR detection of integration of (i) the pCAM-BSD-KOPfCK1 plasmids at the 5’ and 3’ flanks of the insert, (ii) the wild-type locus and (iii) the episome, various primers combinations were used (see Fig 1) to amplify products from genomic DNA. The following primers were used: Primer 1: GGATCCATGGAAATTAGAGTGGCAAATAAATATG; Primer 2: TATTCCTAATCATGTAAATCTTAAA; Primer 3: CAATTAACCCTCACTAAAG; Primer 4: GCGGCCGCTTAATTTTGCTTTACTCTTCCTTC. Primers 1 and 4 correspond to *pfck1* sequences, while primers 2 and 3 correspond to pCAM-BSD vector sequences flanking the insertion site. For detection integration of the pCAM-BSD-CK1GFP and pCAM-BSD-CK1His plasmids, the following combination of primers was used (see S2 Fig for numbering): Primer 1: GCCCTTGGATATGTTCTCATG and primer 4: CAATTAACCCTCACTAAAG. To detect the episome, a combination of primers 2 and primer 3 was used. To detect the wild type locus, primers 1 and 4: CAC AAT TTT AGA AAA ATA ACC ATG C were used. Primer 1 anneals to the PfCK1 coding sequence and primer 4 anneals to the PfCK1 3’UTR (see S1 Fig).

### Southern blotting

Genomic DNA was obtained as follows: parasite pellets obtained by saponin lysis were resuspended in PBS and treated with 150 μg/ml proteinase K and 2% SDS at 55°C for 2 hours. The DNA was precipitated with ethanol and 0.3 M sodium acetate after phenol/chloroform/isoamyl alcohol (25:24:1) extraction. The DNA was digested respectively with NdeI for pCAM KOCK1 transfected plasmids and with Hind III for parasites transfected with pCAM-BSD-CK1-GFP/His.

### Immunoprecipitation

Immunoprecipitation of PfCK1 from parasites extracts or culture supernatants was performed using a rabbit anti CK1 serum (dilution 1:500) and a pre-immune serum (dilution 1:500) as a control. Parasite extracts preparation and immunoprecipitation were performed as described previously [17]. Immunoprecipitation of CK1-GFP was performed according to the GFP-Trap-A Kit (Chromotek) manufacturer’s instructions. Briefly, transgenic or 3D7 parental parasites were harvested after saponin lysis. The pellets were resuspended in cold lysis buffer (30mMTris pH 7.5, 150 mM NaCl, 0.5mMEDTA, 0.5% NP40, 1 mM PMSF, Roche Proteases inhibitors), incubated on ice and briefly sonicated for 3 seconds. The suspension was centrifuged at 14,000 rpm at 4°C for 30 min. The supernatant was applied on GFP-Trap beads for 2 hours at 4°C, and the beads were washed 3 times with dilution buffer (same buffer than above without NP40). Laemmli buffer was added to the beads and boiled for 3 min. The supernatant was loaded on a 12% SDS gel for western blot and on 10% gel for mass spectrometry analysis. Immunoprecipitation was performed as well on supernatants of synchronized cultures (early trophozoites) from transgenic or 3D7 parental parasites. Immunocomplexes were analyzed either by western blot or kinase assay.

### Western blotting

20 μg of 3D7 parasite extracts from asexual stages and gametocytes were prepared as described previously and analyzed on 12% SDS gels. Culture supernatants from synchronized transgenic parasites or 3D7 parental parasites were concentrated on Centricon tubes (Millipore) and applied onto SDS gel for western blot analysis. Anti-PfCK1 western blot was performed using a rabbit immunopurified antibody raised against the PfCK1-derived peptide LENKNRFDQTADQGC (Biogenes; 1:250 dilution) followed by a secondary anti rabbit antibody conjugated to peroxidase (1:1000 dilution). A loading control was obtained with a rabbit 2-cys-peroxyredoxin antibody (1:4000 dilution). Anti-GFP western blot was performed with a mouse anti GFP antibody (Roche; 1:500 dilution). Anti-PfCK2α western blot was performed with a rabbit immunopurified antibody (Biogenes; dilution 1/200) [22]. Anti-PfNapL western blot was performed with a rabbit anti-PfNapL serum (dilution 1/4000) provided by A. Sharma [23]. Anti-PfRON3 western blot was performed with a rabbit antibody (1:200 dilution) provided by T. Tsuboi [24].

### Kinase assays

Kinase assays were performed as described previously [17]. Culture supernatants (5μl) or 500ng of GST-CK1 or GST-CK1K38M were used in a standard kinase reaction using 3μg of substrate: α-casein (Sigma), CK1 peptide substrate (Santa Cruz), GST-PfAlba4 [25], His-PfNapL, His-PfNapS and His-Pf RON3. GST-PfAlba4, His-PfNapL/His-PfNapS and His-PfRON3 expression plasmids were provided by A. Scherf, A. Sharma and T. Tsuboi respectively.

### Immunofluorescence

Immunofluorescence of mixed asexual cultures or synchronized cultures was performed as described previously using thin smears of parasite cultures [17]. Slides were stained by successive incubations with a rabbit anti-CK1 serum or pre-immune serum (1:500 dilution) and with a rhodamine-conjugated anti-rabbit antibody (1:1000 dilution). CK1-GFP was visualized by indirect immunofluorescence using a mouse anti-GFP monoclonal antibody (Roche; 1:500 dilution) followed by an anti-mouse FITC conjugated antibody (dilution 1/1000). PfCK1-GFP/PfRON3 co-localization was investigated using the same anti GFP antibody described above together with a rabbit anti-PfRON3 serum from T. Tsuboi (1:400 dilution). The slides were then washed and mounted with an anti-fading solution containing DAPI. Labelled specimens were examined with a Zeiss Axioscope microscope in combination with an Orca Digital camera.

For immunofluorescence assays on isolated CK1-GFP merozoites, free merozoites were prepared as previously described [26]. Briefly, mature CK1-GFP parasites were synchronized with heparin, magnet purified (VarioMACS, Milteny Biotech) and allowed to recover at 37°C for 5-6h in the presence of 10μM E64 (Sigma). Parasites were resuspended in 1.2ml of PBS and filtered through a 1.2 μm Acrodisc 32mm syringe filter. Free merozoites were dried on a thin slide and IFA performed as described above using an anti-GFP antibody (Invitrogen, rabbit, 1:100) and an anti-AMA-1 antibody (rabbit, 1:100) kindly provided by Paul Gilson.

### Pull-down experiments

Recombinant His-PfRON3 was a gift from T. Tsuboi. Recombinant His-PfCK2α was purified as described [22]. Recombinant GST and GST-PfCK1 were purified as described previously for another kinase, Pfnek-1 [17]. 3μg of GST or GST-PfCK1 bound on Glutathione-agarose beads were incubated with 3μg of soluble His-PfRON3 or His-Pf CK2α for 30 min in 20 mM Tris-HCl (pH 7.5), 0.2 M NaCl, 0.1% Nonidet P40 (IGEPAL), and 10% glycerol at 4°C. The tubes were rotated on a wheel at 4°C for 1 h. After centrifugation, the beads were recovered and washed 4 times in the same buffer, resuspended in Laemmli buffer, boiled and loaded on a SDS gel to analyze the bound material either by Coomassie blue staining or by western blot using an anti-His antibody.

### Mass spectrometry

Samples (immunoprecipitates from 3D7 and CK1-GFP parasites extracts) were separated by SDS-PAGE on a 10% polyacrylamide gel, which was then stained with Biosafe Coomassie (Biorad) and destained in H_2_O. Each SDS-PAGE gel lane was entirely sliced and proteins were digested in-gel using trypsin. Briefly, samples were reduced in 10 mM dithioerythritol (DTE), alkylated in 55 mM iodoacetamide (IAA), and gel pieces were dried. Digestion was performed overnight at 37°C using modified Mass Spectrometry grade trypsin (Trypsin Gold, Promega) at a concentration of 12.5 ng/μl in 50 mM Ammonium Bicarbonate and 10 mM CaCl_2_. Tryptic peptides were extracted from gel slices, dried and resuspended in 2% ACN/0.1% FA for LC-MS-MS analysis. Mass spectrometry analysis was performed on a LTQ Orbitrap XL (Thermo Fischer Scientific) equipped with a Nanoacquity system (Waters). Peptides were trapped on a homemade 5μm 200 Å Magic C18 AQ 0.1 x20 mm pre-column and separated on a home-made 5 μm 100 Å Magic C18 AQ 0.75 x 150 mm column. The analytical separation was performed through a 53 min gradient of H2O/ACN/FA 97.9%/2%/0.1% (Solvent A) and ACN/H2O/FA 98%/1.9%/0.1% (Solvent B). The gradient was run at a constant flow rate of 250nl/min through the following condition: 0–3 min 100% A; 39 min 70%A-30%B; 53 min 100%B. The Mass Spectrometer was operated in an information-dependent mode where the ten most intense precursor ions from each MS survey scan were selected for further CID fragmentation and then excluded for the following 30 seconds. Normalised collision energy of 35% was used for CID fragmentations.

### Data analysis

Raw data were analysed using Maxquant 1.1.1.36 [27] with its internal search engine Andromeda [28]. Fragmentation spectra were searched against the Plasmodium database (PlasmoDB version 6.4–5446 sequences). Maxquant generated the decoy database containing common contaminant proteins and the reversed sequences. A threshold of 1% false discovery rate (FDR) was fixed at the peptide and protein level. Mass spectra were searched with an initial mass tolerance of 6 ppm in MS mode and 0.5 Da in MS/MS mode. Up to two missed cleavages were allowed and carbamidomethylation was set as a fixed modification. Oxidation (M), phosphorylation (S, T, Y) and acetylation (Protein N-term) were considered as variable modifications. A minimum of six amino acids was required as peptide length and at least one (unique + razor) peptide was required for protein identification. Razor peptides are non-unique peptides assigned to the protein group with the most assigned peptides. Reverse and contaminant sequences were removed and proteins with a posterior error probability (PEP) lower than 0.1 were accepted for further analysis. Label-free quantification (LFQ) was performed with razor and unique peptides, using only unmodified and oxidized (M) and acetylated (Protein N-term) peptides. A minimum of one ratio count was required to quantify proteins. The option “Match between runs” was enabled, in which the maximal retention time window was set to 2 minutes. In Perseus, a tool from the Maxquant suite, missing values were imputed with random numbers from a normal distribution with a width of 0.3 and a shift of 1.8 (29). The LFQ intensities (normalized intensities) were compared between CK1 GFP and 3D7 immunoprecipitates, using a statistical Welsch t-test. A permutation-based false discovery rate (FDR) has been applied for multiple testing. The threshold in order to significantly identify interaction partners is based both on the t-test p-values and the ratios. A 0.1 FDR value and a S0 value of 0.5 have been applied. [29,30]. Graphs were obtained with homemade programs written in the R environment [http://www.r-project.org/[31]].

## Results

### Expression pattern of PfCK1

The expression pattern of PfCK1 during the asexual erythrocytic cycle of wild-type parasites was investigated by immunoblot and immunofluorescence analyses using a rabbit antibody directed against a PfCK1-derived synthetic peptide (see Materials and Methods). Western blot analysis of synchronised parasite populations detected a 40 kDa band (close to the predicted 37 kDa molecular mass of PfCK1) in rings, and a stronger signal in trophozoites and schizonts, while the loading control (2-Cys-peroxiredoxin) gave a similar signal in all lanes (Fig 1A); the 40 kDa band was not seen with the pre-immune serum (not shown). This is in agreement with mRNA steady-state levels detected by microarray analysis available on PlasmoDB, and with a previous study [16]. The 40 kDa band was also present in extracts from gametocytes (Fig 1A, right). The anti-PfCK1 antibody, but not the preimmune serum, was able to immunoprecipitate the 40 kDa band (not shown), as well as a casein kinase activity (Fig 1B) from mixed-stage parasite extracts, consistent with the results of experiments performed with parasite proteins that had been affinity-purified on a mammalian CK1 inhibitor [16].

We showed in the context of our kinome-wide reverse genetics study [6] that the *pfck1* gene is refractory to disruption, but can be modified by His_4_ C-terminal tagging, indicating that PfCK1 function is likely essential for parasite proliferation in erythrocytes (see [6] for a summary of the data, and S1A and S1B Fig for locus disruption and GFP/His-tagging strategies, respectively, and for genotyping data). In order to generate an additional tool for monitoring PfCK1 expression and investigating the cellular function of the enzyme, we generated a parasite line expressing PfCK1 fused at its C-terminus with the Green Fluorescent protein (GFP; see S1B Fig for the locus tagging strategy and genotyping of the transgenic line). Western blot analysis of parasite extracts using the anti-PfCK1 antibody showed the expected ~26 kDa shift (from 37 to 63 kDa) in molecular weight between the wild-type and the transgenic line (Fig 1C top left panel). Accordingly, western blot using an anti-GFP antibody detected the 63 kDa band in extracts from transgenic parasites, but gave no signal with extracts from wild-type parental 3D7 parasites (Fig 1C, top right panel). Immunoprecipitates of extracts from transgenic, but not from wild-type parasites, using an anti-GFP antibody contained casein kinase activity (Fig 1D). Likewise, western blot analyses (Fig 1E) and kinase assays following immunoprecipitation (Fig 1F) using an anti-His antibody confirmed that the PfCK1-His transgenic line [6] does indeed express an active His-tagged version of the enzyme.

**Fig 2:**
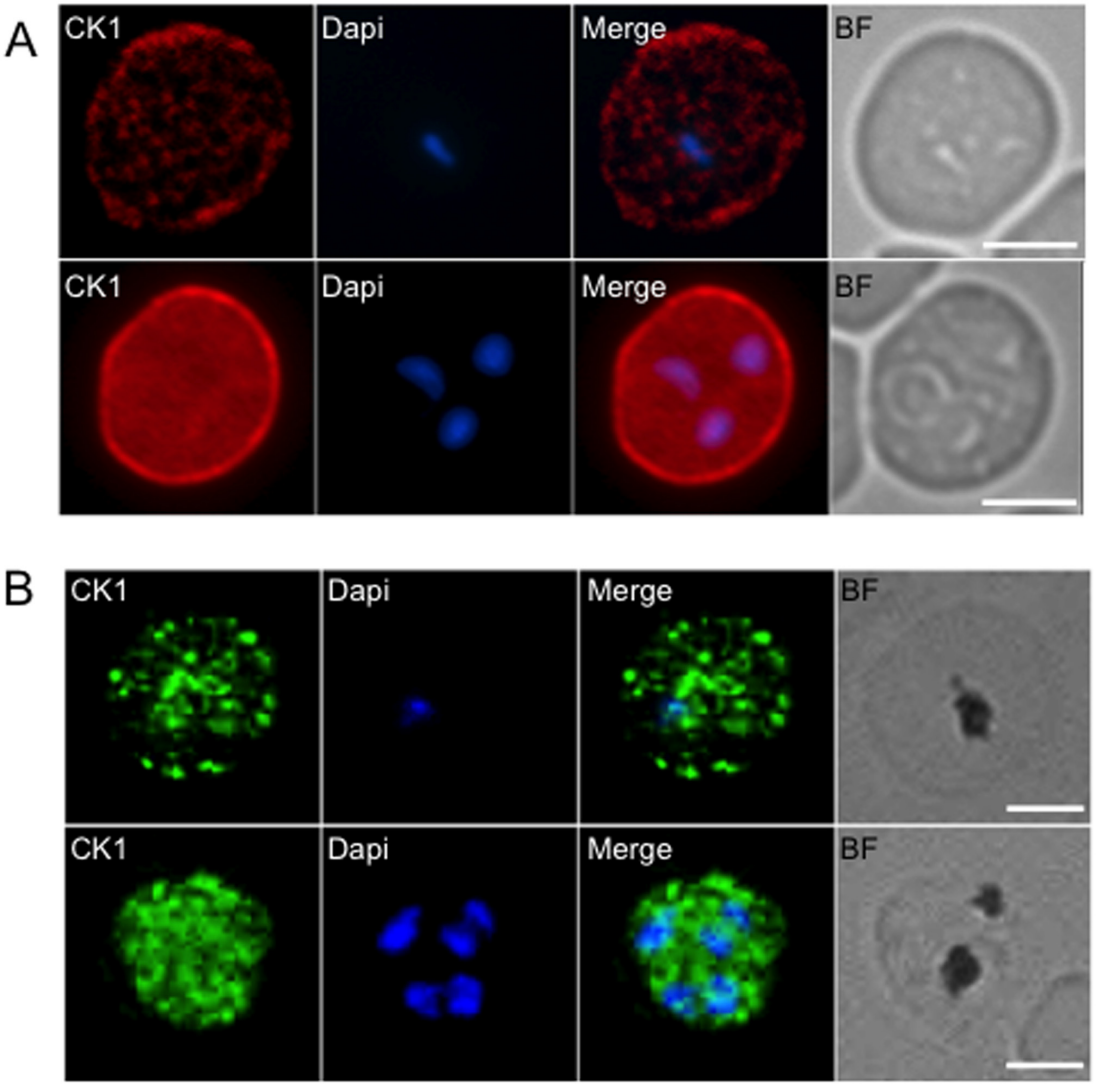
PfCK1 immunofluorescence assay on wild-type parasites and transgenic parasites expressing a GFP-tagged PfCK1. (A) 3D7 parasites were examined by immunofluorescence using an anti-PfCK1 serum labelled with rhodamine. DAPI was used to stain the nucleus and the scale bar represents 10 μm. A pre-immune serum used as a negative control did not yield any signal (not shown). (B) An erythrocyte culture infected with synchronised parasites expressing GFP-tagged PfCK1 from the endogenous locus was examined by immunofluorescence using an anti-GFP antibody. DAPI was used to stain the nucleus the scale bar represents 10 μm. As a negative control wild-type 3D7 parasites stained with the same anti-GFP antibody and did not yield any signal (not shown).

Immunofluorescence assays using the anti-CK1 antibody revealed a strong signal associated with the host erythrocyte cell surface during early stages of infection (rings and young trophozoites) (Fig 2A). No fluorescence was detected with preimmune serum (data not shown). This suggests that a significant fraction of the PfCK1 pool is targeted to the red blood cell membrane, and that this occurs in the early stages of infection. This was confirmed by IFA using the transgenic line expressing GFP-tagged PfCK1 and an anti-GFP antibody (Fig 2B). In mature trophozoites, the staining becomes associated with the parasite itself, with concomitant decrease of the signal associated with the erythrocyte membrane, and in the final stage of infection (segmenters), most of the signal is concentrated into a single dot for each merozoite (see below).

### Interactomics of PfCK1

**Fig 3:**
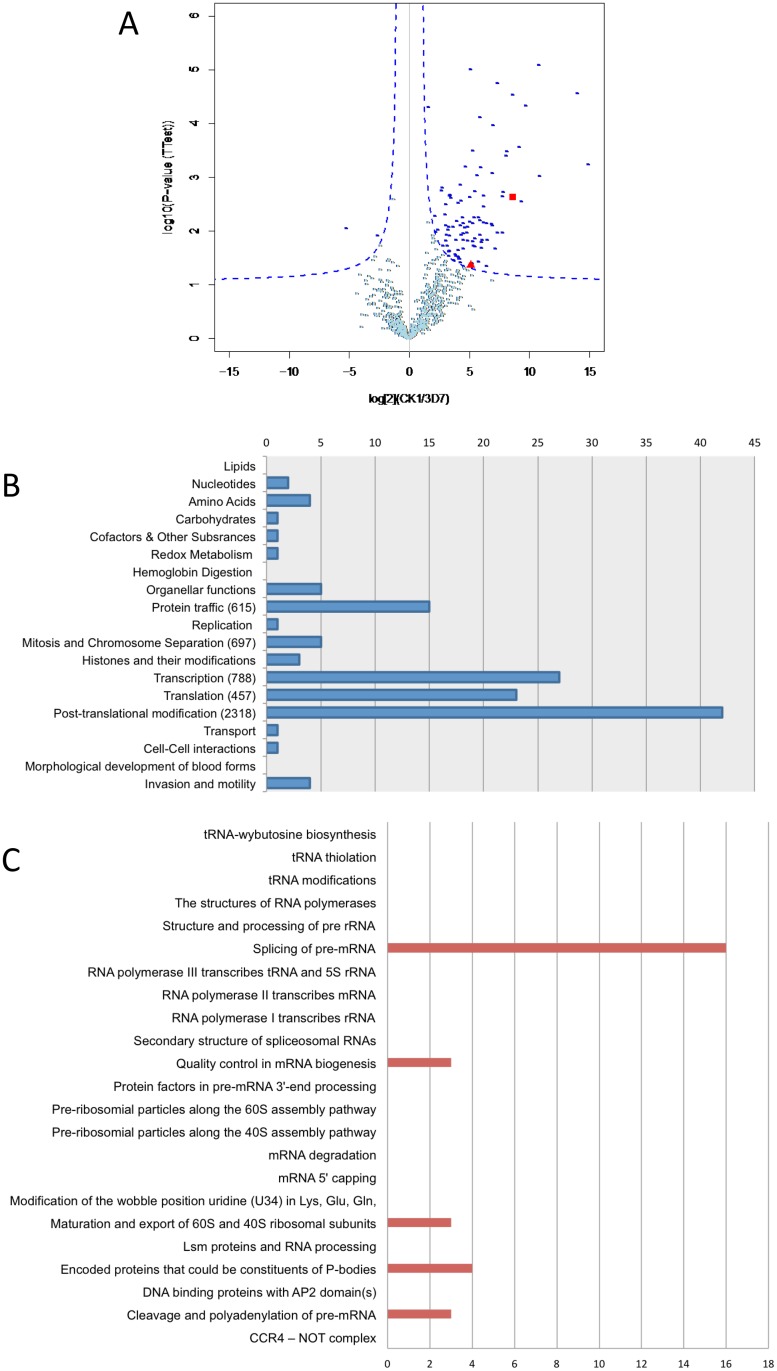
Label-free quantitative analysis of PfCK1 interacting proteins. Volcano plot representing the logarithmic ratio of protein LFQ intensities in the CK1/3D7 experiments plotted against negative logarithmic p-values of the *t* test performed from triplicates (FDR threshold = 0.1, S0 = 0.5). A hyperbolic curve separates specific CK1-interacting proteins (dark blue dots) from background (light blue dots). PfCK1 and PfRON3 are highlighted with a red square and a red triangle respectively. (B) Distribution of potential PfCK1 interactors across metabolic processes. Histogram representing the number of potential PfCK1 interactors distributed among the various metabolic processes described in the Metabolic Pathways of Malaria Parasites website (http://sites.huji.ac.il/malaria/). The total number of proteins in the most represented pathway is represented in brackets. (C) Distribution of potential PfCK1 interactors across specific pathways in the transcription process. The histogram represents the number of potential PfCK1 interactors across the various pathways present in the “Transcription” process.

The distribution of PfCK1 in numerous compartments within the parasite itself and in the host cell suggests multiple functions, in line with the pleiotropic role of CK1 isoforms in other systems. In order to gain insight into cellular processes that may be under PfCK1 control, we performed an interactomics study by analysing the protein content of immunoprecipitates obtained with anti-GFP antibodies from extracts of transgenic parasites expressing GFP-tagged PfCK1 from the cognate locus, using extracts from wild-type 3D7 parasites as a negative control. We had implemented this approach in another study with PfCK2, which allowed us to infer a function in chromatin assembly for the latter enzyme [25]. GFP-PfCK1 immunoprecipitates were run on two polyacrylamide gels; one of these was used for transfer and western blotting to verify the presence of PfCK1 in the immunoprecipitate from transgenic, but not from wild-type, parasites (not shown). The other gel was divided into several slices, which were then processed for proteomics analysis (not shown) (see Materials and Methods). The data from three IP experiments were analysed by MaxQuant (see S1 Table for a full list of quantified proteins), and results were displayed as a volcano plot (Fig 3A). As expected, PfCK1 was identified in the immunoprecipitate. All 97 proteins identified as high probability interactors based on threshold values (see Materials and Methods) in the volcano plot analysis were listed (S2 Table) and matched against the pathways described in the “Metabolic pathways of Malaria Parasites” website (http://mpmp.huji.ac.il; analyses were performed with the September 2013 version). As expected from the pleiotropic nature described for CK1 orthologues in many eukaryotic systems, proteins involved in a large number of processes were recovered (Fig 3B). If we exclude the “post-translational modification” process, which recovered the maximum number of proteins due to the large number of phosphoproteins included in this category, the most represented process was “Transcription”, which includes a number of different specific pathways (Fig 3C). Interestingly, most (16/27) of the proteins associated with the “Transcription” process belong to the “mRNA splicing pathway”, strongly suggesting that PfCK1 is associated with the spliceosome (Fig 3C and S2 Fig).

We focussed validation experiments on the rhoptry protein PfRON3 (Plasmodb ID: PFL2505c / PF3D7_1252100, in view of its major role in invasion [24,32]; this protein is listed in the high-probability interactors (S2 Table). We also selected two potential interactors that appeared in the full list of proteins co-purifying with PfCK1 (S1 Table) but did not make the threshold value on the volcano plot (S2 Table): (i) PfCK2α (Plasmodb ID: PF11_0096 / PF3D7_1108400), as we had previously shown in a reciprocal experiment that PfCK1 co-precipitates with a PfCK2 regulatory subunit (PfCK2β) [25]. Consistently, catalytic (α) and regulatory (β; Plasmodb ID: PF13_0232 / PF13_0232) subunits of PfCK2 are in the list of proteins that were co-immunoprecipitated with PfCK1 (S1 Table); (ii) the nucleosome assembly protein PfNapL (Plasmodb ID: PFL0185c / PF3D7_1203700), which we had previously shown to also be associated with PfCK2α [25].

**Fig 4:**
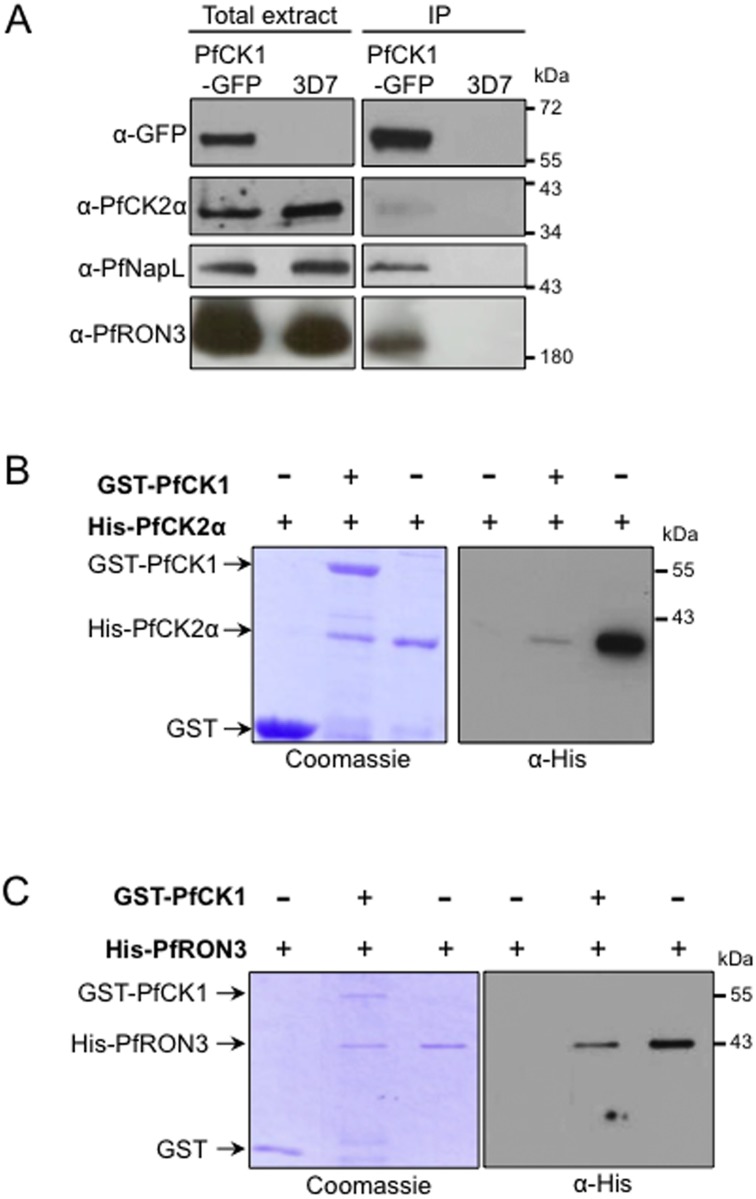
Interaction between PfCK1 and PfNapL, PfCK2a or PfRON3. **(A) Interaction between native PfCK1 and PfNapL, PfCK2a or PfRON3.** Immunoprecipitation was performed on protein extracts from transgenic parasites expressing GFP-tagged PfCK1 and from wild-type 3D7 parasites using GFP-trap beads. Detection of GFP, PfCK2a, PfNapL and PfRON3 was then performed by western-blot on the immunoprecipitates using the *ad hoc* antibodies. Lane 1: total extracts from PfCK1-GFP parasites; lane 2: total extracts from 3D7 WT parasites; lane 3: immunoprecipitates from PfCK1-GFP parasites; lane 4: immunoprecipitates from 3D7 WT parasites. **(B) Recombinant PfCK1 and PfCK2α interact *in vitro*.** GST-PfCK1 was incubated with His-PfCK2α and complexes containing the CK1 GST-tagged protein were then purified using glutathione agarose beads. The His-tagged proteins were detected by Western blot analysis using an anti-His antibody and the corresponding Coomassie blue-stained gels are shown. Lane1: Bound material after incubation of GST agarose beads with soluble His-PfCK2α; lane 2: bound material after incubation of GST-PfCK1 agarose beads with soluble His-PfCK2α; lane 3: soluble His-PfCK2α control. **(C) Recombinant PfCK1 and PfRON3 interact *in vitro*.** GST-PfCK1 was incubated with His-PfRON3 and complexes containing the CK1 GST-tagged protein were then purified using glutathione agarose beads. The His-tagged proteins were detected by Western blot analysis using an anti-His antibody and the corresponding Coomassie blue-stained gels are shown. Lane 1: bound material after incubation of GST agarose beads with soluble His-PfRON3; lane 2: bound material after incubation of GST-PfCK1 agarose beads with soluble His-PfRON3; lane 3: soluble His-PfRON3 control.

**Fig 5:**
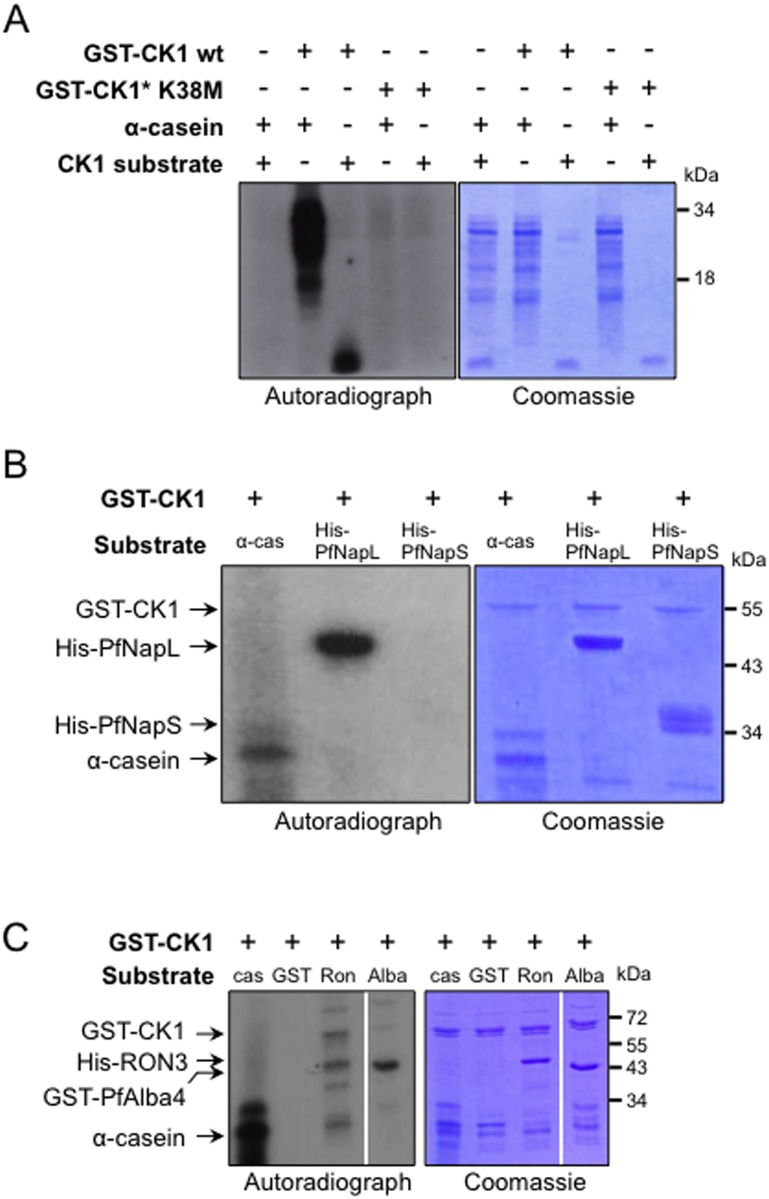
Kinase activity of recombinant PfCK1 on potential interactors. (A) Autoradiograms and Coomassie blue stained gels of *in vitro* standard kinase reactions. Assays were performed with 500 ng of recombinant GST-PfCK1wild-type or with GST-PfCK1 K38M (“kinase dead mutant”). Both kinases were incubated with 3 μg of α-casein, or CK1 peptide substrate. GST-PfCK1 wt shows activity towards these 2 substrates whereas no signal was obtained with the control mutant. Lane 1: α-casein + CK1 substrate; lane 2: GST-PfCK1 WT + α-casein; Lane 3: GST-PfCK1 WT + CK1 substrate peptide; lane 4: GST-PfCK1[K38M]+ α-casein; lane 5: GST-CK1[K38M] + CK1 peptide substrate. (B) Phosphorylation of α-casein and PfNapL, but not PfNapS, by GST-PfCK1. Lane 1: GST-PfCK1 + α-casein; lane 2: GST-PfCK1 + His-PfNapL; lane 3: GST-PfCK1 + His-PfNapS. (C) Phosphorylation of PfRON3 and PfAlba4 by GST-PfCK1. Lane 1: GST-PfCK1+ α-casein; lane 2: GST-PfCK1 + GST; lane 3: GST-PfCK1 + His PfRON3; lane 4: GST-PfCK1 + GST-PfAlba 4.

**Fig 6:**
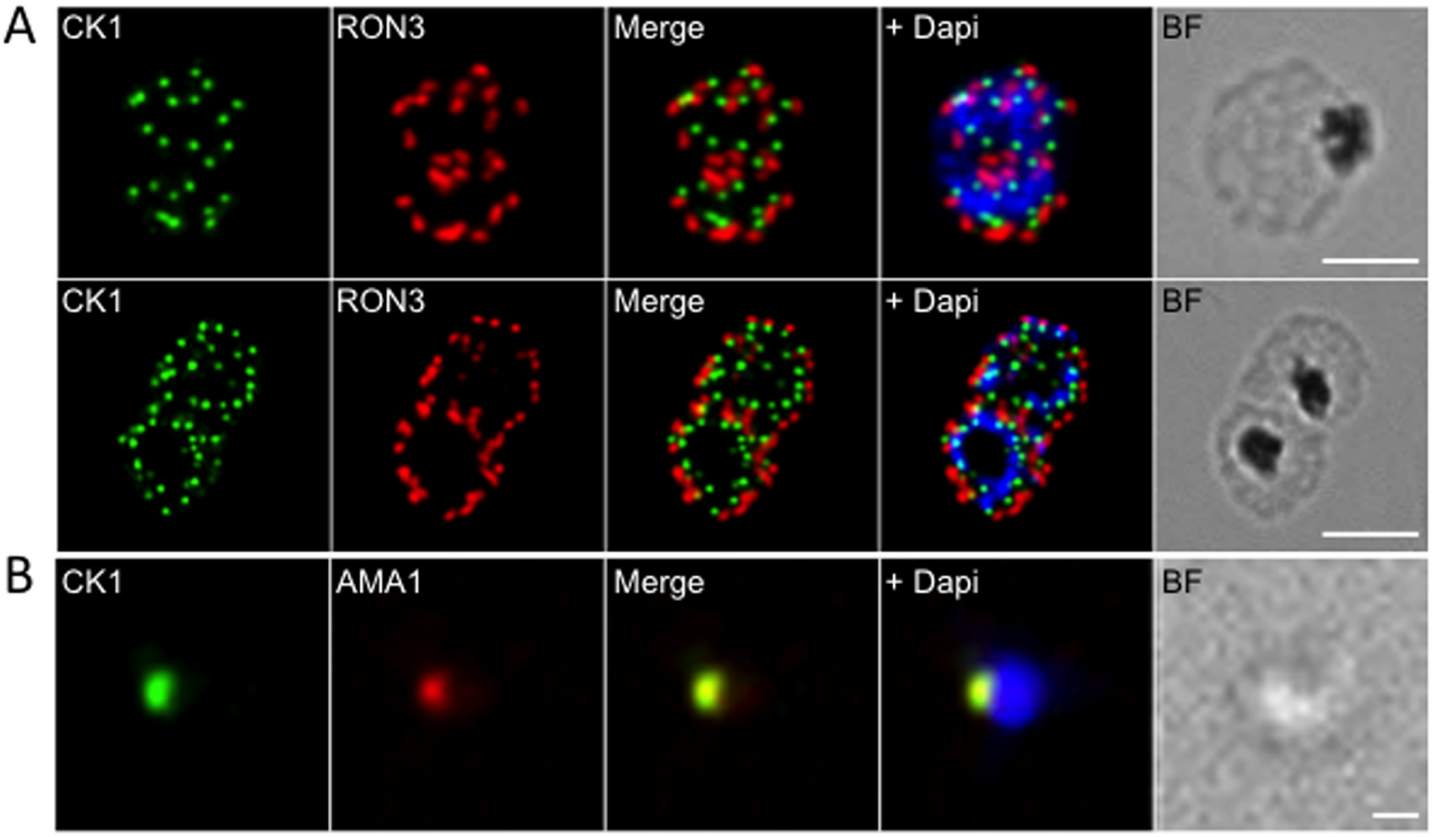
Localisation of PfCK1 in micronemes. (A) Localisation of PfCK1 and PfRON3 in schizonts and segmenters. DAPI was used to stain the nucleus and the scale bar represents 10μm. (B) Localisation of PfCK1 and AMA-1 in merozoites. DAPI was used to stain the nucleus and the scale bar represents 1μm.

For these three proteins, we performed western blot analyses of GFP-PfCK1 immunoprecipitates with cognate antibodies (Fig 4A). In all three cases, we detected the target protein in the immunoprecipitates obtained with extracts from transgenic parasites expressing GFP-tagged PfCK1 from the endogenous locus, but not from the wild-type parasites used as a negative control. Direct interaction between PfCK1 and PfCK2α, and between PfCK1 and PfRON3, was further documented by pull-down assays using recombinant proteins (Fig 4B and 4C respectively). To determine whether the selected interactors can act as substrates for PfCK1, we performed *in vitro* kinase assays using recombinant proteins. We first verified that recombinant PfCK1 has kinase activity on caseins as expected, using as a negative control a kinase-dead mutant in which Lys38 (a residue required for proper orientation of ATP and hence essential for kinase activity) is replaced with a Met, to ensure that activity is indeed associated with the recombinant enzyme rather than with co-purifying bacterial material (Fig 5A). Recombinant RON3 and PfNapL (but not PfNapS, Plasmodb ID: PFI0930c / PF3D7_0919000) are indeed substrates for recombinant PfCK1 (as is Alba, an RNA-binding protein also present in the putative interactome) (Fig 5B and 5C and S1 Table). Co-localisation immunofluorescence experiments indicate partial overlap of PfCK1 with PfRON3 in schizonts, but the proteins segregate into distinct, close but non-overlapping dots in segmenters (Fig 6A). Since RON3 is localised in the rhoptry bulb [32], the non-overlapping localisation with PfCK1 suggests that the kinase is located in a nearby but distinct secretory organelle, the microneme. Indeed, in isolated merozoites, PfCK1 co-localises exactly with the micronemal protein AMA1 (Fig 6B; see Discussion).

### A pool of PfCK1 is secreted into the culture supernatant

**Fig 7:**
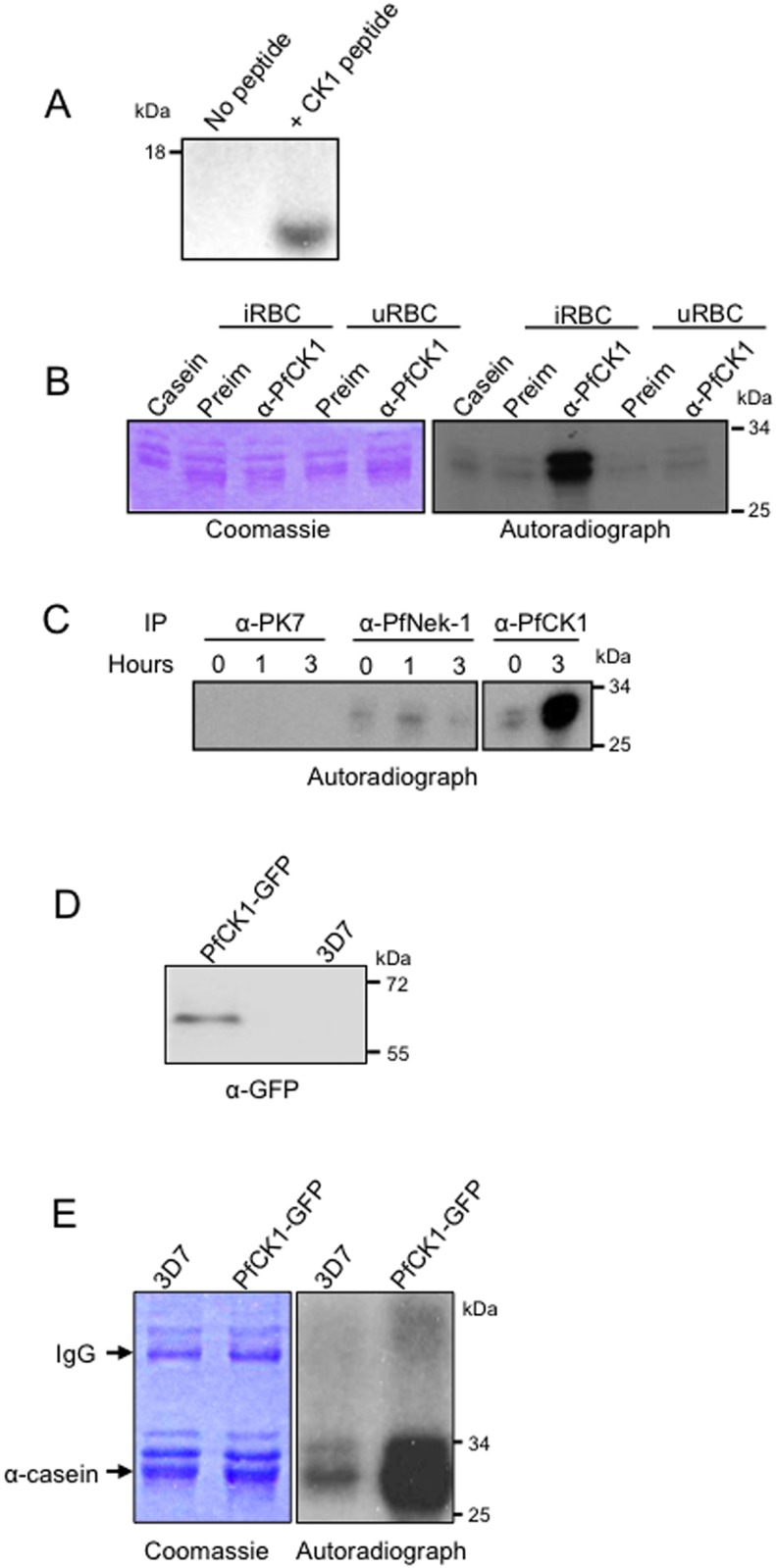
PfCK1 kinase activity in culture supernatants. (A) CK1 peptide phosphorylation by culture supernatants. (B) PfCK1 activity immunoprecipitated from culture supernatants of uninfected (uRBCs) or 3D7-infected (iRBC) red blood cells using an anti-PfCK1 antibody (α-PfCK1). A pre-immune serum (preim) was used as control. (C) Accumulation of casein kinase activity immunoprecipitated from supernatants of synchronised trophozoite cultures using antibodies against PfPK7, PfNek-1 and PfCK1. All three kinases immunoprecipitated from parasite extracts gave strong signals (not shown). (D) Secretion of endogenous GFP-tagged PfCK1. Anti-GFP Western blot showing GFP-tagged PfCK1 in the supernatant of transgenic parasites expressing GFP-tagged PfCK1, but not in supernatants of WT parasites. (E) Kinase activity immunoprecipitated with an anti-GFP antibody from culture supernatants. Lane 1: supernatant from a wild-type 3D7 culture; lane 2: supernatant from a transgenic line expressing GFP-tagged PfCK1.

Interestingly, some CK1 isoforms from other parasites [33], as well as from yeast [34] and mammalian cells [35], have been shown not only to be expressed at the cell surface as “ectokinases” (consistent with PfCK1 antibodies staining the host erythrocyte boundary), but also to be secreted to the extracellular medium. This prompted us to assay culture supernatants for the presence of CK1 activity, using a peptide substrate that is highly specific for CK1 orthologues. Clearly, supernatants of infected erythrocyte cultures contain an activity that is able to phosphorylate the peptide (Fig 7A). To test whether the activity was mediated by secreted PfCK1, the PfCK1 antibody (or a preimmune serum as a negative control) was used in immunoprecipitations (IP) from culture supernatants, and the immunoprecipitates were assayed for casein kinase activity (Fig 7B). The anti-PfCK1 antibody (but not the preimmune serum) was able to pull-down a robust casein kinase activity from infected cell supernatants, but not from the supernatant from uninfected erythrocytes cultures. To rule out the possibility that this may result from non-specific release of parasite content upon schizont rupture, we repeated the IP/kinase assay experiment using synchronous cultures at the early trophozoite stage, and assaying the supernatant 0, 1 and 3 hours after resuspension in fresh medium, long before schizont rupture. The results (Fig 7C) show that casein kinase activity accumulates in the supernatant under these conditions. As an additional control, we performed similar IP/kinase assays using antibodies directed against protein kinases known to be intracellular, such as PfPK7 [36,37] and Pfnek-1 [17]. Although control immunoprecipitates from parasite extracts obtained with the PfPK7 and Pfnek-1 antibodies were verified to indeed contain strong kinase activity as expected (not shown), accumulation of kinase activity in the culture supernatant was detected neither in PfPK7 nor in Pfnek-1 immunoprecipitates, while the PfCK1 IP yielded a strong signal (Fig 7C).

To further ascertain that PfCK1 is secreted into the culture medium, we used the parasite line expressing GFP-tagged PfCK1 from the cognate locus (see S1B Fig for the strategy and genotyping of this line). The 63 kDa PfCK1-GFP band was readily detectable not only in parasite extracts (Fig 1C), but also in parasite supernatants from the transgenic line only, but not from wild-type parasites (Fig 7D). In line with these data, we were able to IP casein kinase activity with the anti-GFP antibody from both parasite extracts (Fig 1D) and medium supernatant (Fig 7E) from transgenic, but not wild-type, parasites. Similar IP results were obtained using the transgenic line expressing His-tagged instead of GFP-tagged PfCK1 (data not shown).

## Discussion

### Subcellular localisation

CK1 isoforms participate in multiple cellular processes in eukaryotic cells, and the diverse sub- and extra-cellular localisations that we document here, together with the complex putative interactome, indicate that this holds true for PfCK1, the only member of the CK1 group in *P*. *falciparum*. In the yeast *Saccharomyces cerevisiae*, the five CK1 isoforms associate with discrete cellular compartments, which is crucial for their respective functions [14]. One would predict that if the plasmodial enzyme also plays a role in multiple processes, it must have wide-ranging subcellular distribution, and this is indeed what we document here (Figs 2 and 6). The cellular distribution of PfCK1 appears to be dynamic, with a pool of enzyme being exported to the host erythrocyte periphery at the early stage of infection, and subsequently detectable mostly within the parasite in late trophozoites and schizonts. A punctate pattern emerges in segmenters, with one PfCK1 dot in each developing merozoite, adjacent to the rhoptry marker RON3 [32] (Fig 6A). In line with this observation, PfCK1 colocalises with AMA-1 (Fig 6B), a protein of micronemes that is released at the very early stages of invasion [38,39]. It is tempting to speculate that PfCK1 is released from micronemes very early in the invasion process, which may explain its presence on the red blood cell membrane in early stages of the infection. Since soluble PfCK1 accumulates in the supernatant of synchronised parasites at the trophozoite stage (Fig 7C), however, possible secretion during microneme release (if it happens) is clearly not the whole story (see below). It is of interest to mention that the related Apicomplexan parasite *Toxoplasma gondii* expresses two CK1 isoforms, with TgCK1α being cytosolic and TgCK1β being associated via its C-terminus with the plasma membrane [40]. Localisation of the yeast CK1 homologue Yck2p to the membrane is mediated by palmitoylation [41]; PfCK1 was not detected in a proteome-wide search for palmitoylated proteins [42] which revealed that palmitoylation is pervasive in malaria parasites. Nevertheless, it cannot be excluded that such a modification occurs in a transient manner and thus may have been missed in this analysis. Indeed, our data indicate that membrane association occurs only in early stages of the asexual replication cycle.

### Interactomics

Likewise, the complexity of the interactome concurs to suggesting an involvement in a number of pathways. The specificity of the IP/MS approach we implemented here is illustrated by the fact that very little overlap was observed between the proteins recovered from the PfCK1 immunoprecipitates (this study) and those associated with PfCK2 [25], with only 2 proteins from the mRNA splicing pathway co-precipitating with PfCK2 and 16 with PfCK1; conversely, CK2 associated with 12 proteins in the nucleosome assembly and regulation pathways [25]. Interestingly, protein involved in chromatin assembly, i.e. NapS, Nap L (see above), and histones H2A, H2a variant, H3 and H4, were recovered in the PfCK1 immunoprecipitates (although of these, only histone H4 made it through the stringent threshold for inclusion in the high-probability interactors (S2 Table)). In contrast none of the proteins that regulate chromatin regulation that copurified with PfCK2 (e.g. chromodomain or Smarca proteins) were found in the PfCK1 precipitates. This suggest that PfCK1 may have a function during chromatin assembly, but not in the regulation of gene expression through chromatin modification, a hypothesis whose validation clearly needs additional work. In addition to a predicted role of PfCK1 in mRNA splicing, invasion and chromatin structure, and based on co-precipitating proteins as presented in the Results section, it is noteworthy that two Rab proteins (Rab2 and Rab7) were identified as putative interactors, corroborating earlier studies implicating PfCK1 in vesicular trafficking, notably on the basis of interaction with Rab5C [43,44]. Since Rab5B is myristoylated, it is a possibility that its membrane association may explain why Rab5B was not also detected in our study, since membrane-associated proteins are likely to be under-represented in the extracts used as input material for the immunoprecipitation.

PfCK1 has been repeatedly identified as a phosphoprotein in rings, schizonts and merozoites [5,7,45], with occupied phosphosites at S17, S19 and T313. Auto-phosphorylation of the recombinant enzyme is not apparent ([16]; this study), suggesting that other kinases are likely involved. Some kinases are present in the immunoprecipitates; although the statistics we adopted do not warrant their inclusion in our list of potential interactors (S2 Table), these enzymes may still phosphorylate PfCK1. Such kinases include PfCK2 (see above), and the acidic environment of T313 (RFDQT*ADQEGRV) may be consistent with PfCK2 acting at this site. The other sites (S17 and S19) are, interestingly, located within the glycine triad in subdomain I that mediates orientation of ATP (LGS*GS*FGDIYV). In the cyclin-dependent kinases, phosphorylation in this region (T14-Y15 in human CDK1) is inhibitory [46,47], and a similar mechanism may be operating to regulate CK1 activity. It is relevant to note, however, that phosphorylation of a residue within the glycine triad of PfCDPK1 did not affect the activity of the enzyme [48].

### Export and secretion of PfCK1

Our observation (Fig 2A) that PfCK1 associates with the periphery of the host erythrocyte (likely with the plasma membrane, although this has not been formally established) is consistent with the documented plasma membrane localisation of at least one CK1 isoform in yeast [34] and in *Toxoplasma gondii* [40] (see above), and with ‘ectokinase’ properties of CK1 isoforms in some mammalian cell types [35] and in the parasitic protist *Leishmania* [33]. This is consistent with the intriguing observation that a cell-impermeable inhibitor, Purvalanol B, which has no detectable activity on a panel of human cell lines, kills *P*. *falciparum* with a low micromolar IC50 [49]; PfCK1 was subsequently identified as the major protein binding to immobilised Purvalanol B [50]. We are currently addressing the question of whether the inhibitor exerts its parasitocidal effect through inhibition of surface-exposed PfCK1, without the need to enter the cell—this would have major implications with respect to PfCK1 representing a valuable target for pharmaceutical intervention. Furthermore, PfCK1 is clearly released by trophozoites-infected erythrocytes and accumulates in the culture medium (Fig 7). There is growing evidence that *Plasmodium*-infected erythrocytes secrete signalling molecules. Thus, an unbiased analysis of culture supernatants identified a protein phosphatase as well as PfTKL2, a member of the “Tyrosine kinase-like kinases” group [51]; this was corroborated by an independent study focussed on this enzyme [52]. Secretion of protein kinases to the culture medium has been observed in other parasitic eukaryotes, for example CK1 isoforms of *Leishmania donovani* promastigotes [53], and the catalytic subunit of another kinase, CK2, in the parasitic helminth *Schistosoma mansoni* [54]. What may be the function of these secreted enzymes? In mammals, there is growing evidence that secreted kinases play major roles in the regulation of extracellular elements, for example in the biomineralisation, and may regulate the function or accessibility of receptors with an extracellular ligand-binding domain to their ligands (reviewed in [55]). In a similar perspective, and in view of the importance of cell-cell interactions and communication between cells in the life cycle of malaria parasites, it is attractive to speculate that a function of secreted kinases is to phosphorylate the extracellular domain of proteins, so as to facilitate binding of the merozoite to its next host erythrocyte, or of the infected red blood cell to a range of host cells, such as endothelium (cytoadherence), uninfected erythrocytes (rosetting) and platelets (platelet-mediated clumping) [56]. Another hypothesis is that secreted macromolecules can be delivered to bystander immune or endothelial cells as cargo of microparticles or exosomes; it has indeed been demonstrated that microparticles produced by infected red blood cells can dramatically influence cell surface phenotype and morphology of bystander endothelial cells [57]. Furthermore, evidence that exosomes produced by infected erythrocytes regulate sexual differentiation of parasites residing in nearby red blood cells, pointing to signalling events mediated by secreted macromolecules [58]. The fact that active PfCK1 can be recovered from culture supernatants by direct immunoprecipitation (Fig 7) suggests that at least a fraction of secreted PfCK1 is not packaged inside microparticles or exosomes. In view of the absence of signal and PEXEL motifs, the mechanisms pertaining to secretion of PfCK1 remain to be elucidated. PfCK1 co-purifies with several Rab proteins (Rab2 and Rab7 as determined in this study, and Rab5B as revealed previously (41, 42)), and a potential role for Rab proteins in the export and secretion of CK1 warrants further investigations. For example, it would be of interest to determine whether myristoylated Rab5B mediates trafficking of CK1 to the erythrocyte membrane. Finally, an autocrine/paracrine loop mechanism, whereby secreted kinases are required in *trans* for survival of parasites in the same culture, cannot be ruled out. We are implementing transwell-based experiments to explore this intriguing possibility.

## Supporting Information

S1 Fig Genetic modification of the *pfck1* locus in *P*. *falciparum* parasites. **(A) Attempt at knocking-out the *PfCK1* gene.** *pfck1*gene disruption strategy and Southern Blot analysis. The size of the bands obtained after NdeI digestion is indicated in the diagram. **(B**) **PfCK1-GFP and –His tagged parasites.** Illustration of the C-terminus tagging strategy and Southern Blot analysis. The size of the bands obtained after HindIII digestion is indicated in the diagram.
(TIF)

S2 Fig Pf CK1 potential interactors involved in the mRNA splicing pathway. In the transcription pathway, most of the potential interactors found belong to the splicing mRNA in *Plasmodium falciparum* http://priweb.cc.huji.ac.il/malaria/maps/splicing.html. U symbolize small ribonucleoproteins binding to specific pre-mRNA sequences. Next to each enzyme there is a pie that depicts the stage-dependent transcription of the enzyme's coding gene. The pie is constructed as a clock of the 48 hours of the parasite cycle, where red signifies over-transcription and green, under-transcription. The accession numbers in red characters highlight the proteins identified as putative PfCK1 interactors in the present study.
(TIF)

S1 Table List of quantified proteins recovered from PfCK1 and 3D7 immunoprecipitates. Columns A to F: Quantification (LFQ = Label Free Quantification) obtained in various biological replicates.
Column G (t-test significance): « + » indicates a p-Value and S0 for differential protein content in sample and control that comply with the threshold requirements (See Materials and Methods). Column H: PEP: Posterior Error Probability (probability of protein identification). Column I: -Log t-test p value: transformation of t-test value for display on the plot (Fig 3A). Column J (t-test Difference): Log [2] CK1/3D7. Column K: Protein IDs = PlasmoDB Accession Number. Column L: Protein Description.
(XLSX)

S2 Table List of PfCK1 interactors obtained by label-free quantitative analysis (post-statistical threshold). The previous and new PlasmoDB accession numbers of proteins identified as potential PfCK1 interactors are indicated.
(PDF)
