# Supplementary figures and images for: Malaria Parasite-Infected Erythrocytes Secrete PfCK1, the Plasmodium Homologue of the Pleiotropic Protein Kinase Casein Kinase 1
Source: PLoS One. 2015 Dec 2;10(12):e0139591. doi: 10.1371/journal.pone.0139591 (PMC4668060; doi:10.1371/journal.pone.0139591)

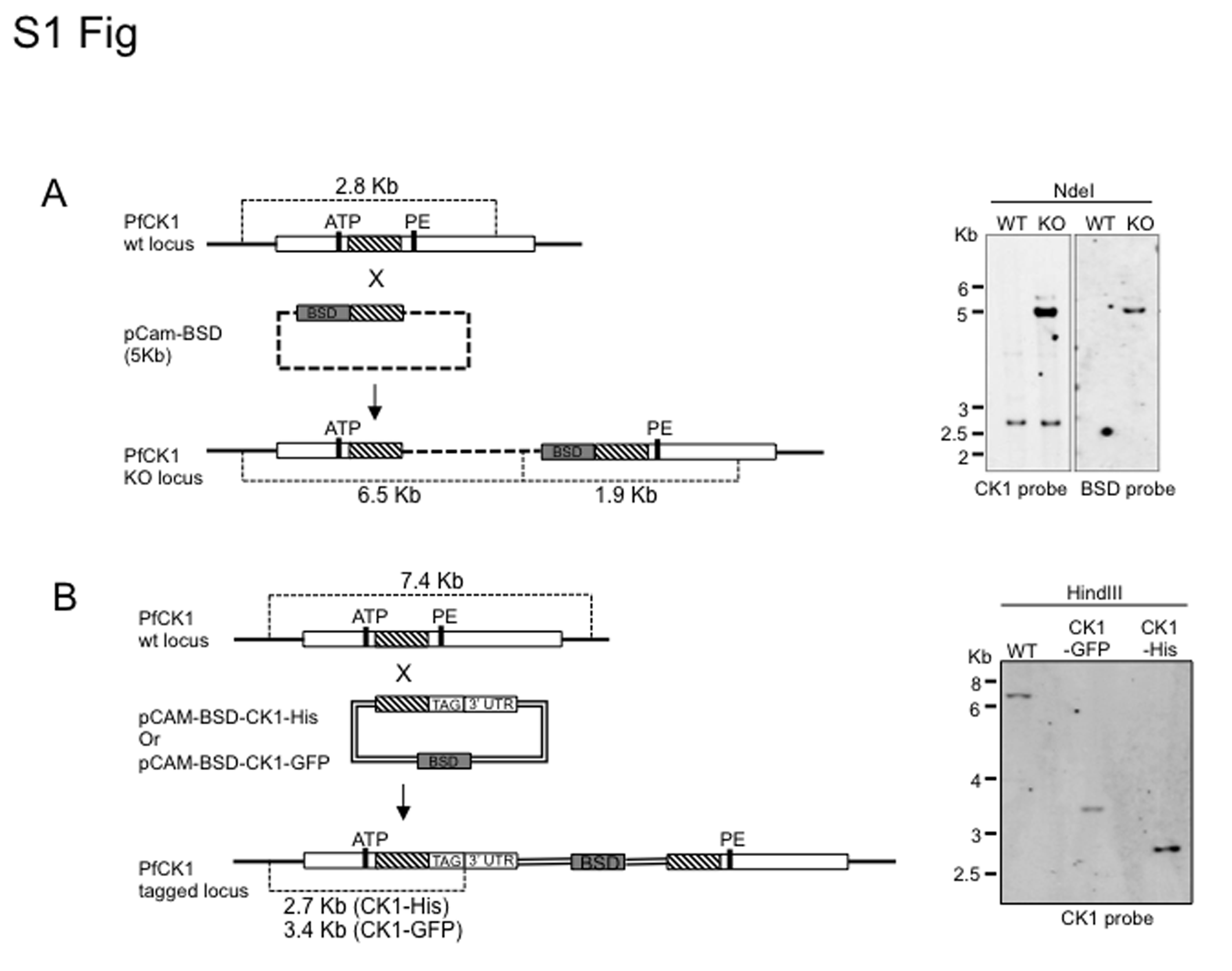

Supplement: S1 Fig — (A) Attempt at knocking-out the PfCK1 gene. pfck1gene disruption strategy and Southern Blot analysis. The size of the bands obtained after NdeI digestion is indicated in the diagram. (B) PfCK1-GFP and –His tagged parasites. Illustration of the C-terminus tagging strategy and Southern Blot analysis. The size of the bands obtained after HindIII digestion is indicated in the diagram. (TIF) [file pone.0139591.s001.tif]

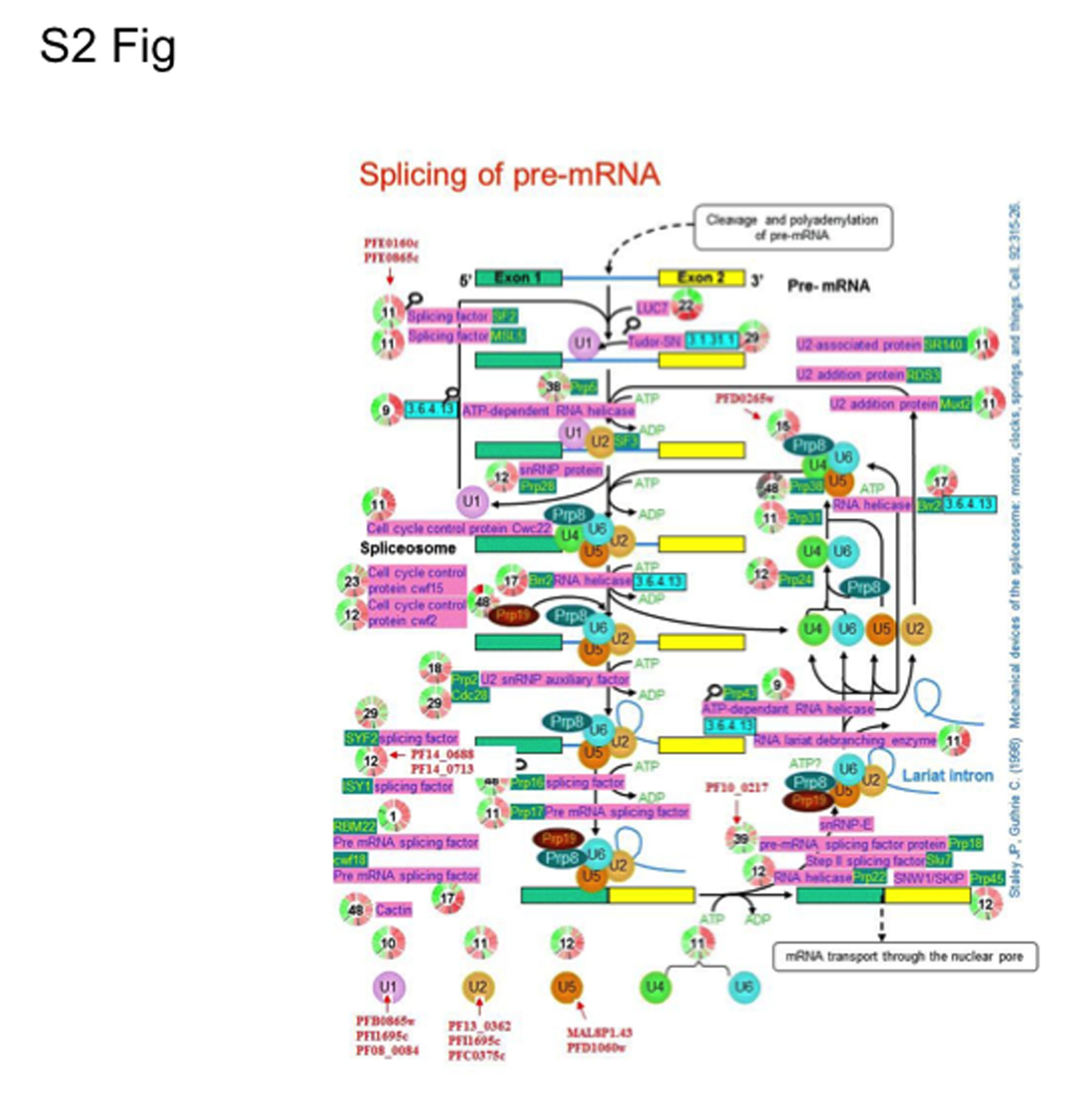

Supplement: S2 Fig — In the transcription pathway, most of the potential interactors found belong to the splicing mRNA in Plasmodium falciparum http://priweb.cc.huji.ac.il/malaria/maps/splicing.html. U symbolize small ribonucleoproteins binding to specific pre-mRNA sequences. Next to each enzyme there is a pie that depicts the stage-dependent transcription of the enzyme's coding gene. The pie is constructed as a clock of the 48 hours of the parasite cycle, where red signifies over-transcription and green, under-transcription. The accession numbers in red characters highlight the proteins identified as putative PfCK1 interactors in the present study. (TIF) [file pone.0139591.s002.tif]
