# Supplementary material for: Malaria Parasite-Infected Erythrocytes Secrete PfCK1, the Plasmodium Homologue of the Pleiotropic Protein Kinase Casein Kinase 1
Source: PLoS One. 2015 Dec 2;10(12):e0139591. doi: 10.1371/journal.pone.0139591 (PMC4668060; doi:10.1371/journal.pone.0139591)
Supplement: S2 Table — The previous and new PlasmoDB accession numbers of proteins identified as potential PfCK1 interactors are indicated. (PDF) [file pone.0139591.s004.pdf]

	New Plasmodb accession number	Previous Plasmodb accession number	Annotation
1	PF3D7_1341300	MAL13P1.209	ribosomal protein L18-2
2	PF3D7_1314700	MAL13P1.75	conserved Plasmodium protein
3	PF3D7_0823200	MAL8P1.40	RNA binding protein
4	PF3D7_0822800	MAL8P1.43	U5 snrnp-specific protein
5	PF3D7_0710600	PF07_0043	60S ribosomal protein L34-A
6	PF3D7_0814200	PF08_0074	DNA/RNA-binding protein Alba
7	PF3D7_0812500	PF08_0086	RNA binding protein
8	PF3D7_1002400.1	PF10_0028	RNA binding protein
9	PF3D7_1004500	PF10_0048	conserved Plasmodium protein
10	PF3D7_1006200	PF10_0063	DNA/RNA-binding protein Alba
11	PF3D7_1011800	PF10_0115	QF122 antigen
12	PF3D7_1022400	PF10_0217	pre-mRNA splicing factor
13	PF3D7_1027800	PF10_0272	60S ribosomal protein L3
14	PF3D7_1005000	PF11_0061	histone H4
15	PF3D7_1110400	PF11_0111	asparagine-rich antigen
16	PF3D7_11136500.2	PF11_0377-a (+2)	acasein kinase 1
17	PF3D7_1145400	PF11_0465	dynamin-like protein
18	PF3D7_1308200	PF13_0044	carbamoyl phosphate synthetase
19	PF3D7_1323100	PF13_0129	60S ribosomal protein L6
20	PF3D7_1330800	PF13_0165	conserved Plasmodium protein
21	PF3D7_1331800	PF13_0171	60S ribosomal protein L23
22	PF3D7_1341200	PF13_0224	60S ribosomal protein L18
23	PF3D7_1342000	PF13_0228	40S ribosomal protein S6
24	PF3D7_1352700	PF13_0273	conserved Plasmodium protein
25	PF3D7_1414800	PF14_0146	ribonucleoprotein
26	PF3D7_1424400	PF14_0231	60S ribosomal protein L7-3
27	PF3D7_0218500	PFB0865w	small nuclear ribonucleoprotein
28	PF3D7_0302000	PFC0100c	regulatory protein
29	PF3D7_0316500	PFC0720w	conserved Plasmodium protein
30	PF3D7_0322900	PFC1020c	40S ribosomal protein S3A
31	PF3D7_0401800	PFD0080c	Plasmodium exported protein (PHISTb)
32	PF3D7_0403700	PFD0180c	CGI-201 protein
33	PF3D7_0405400	PFD0265w	pre-mRNA splicing factor
34	PF3D7_0410800	PFD0535w	conserved Plasmodium protein
35	PF3D7_0414000	PFD0685c	cchromosome associated protein
36	PF3D7_0415900	PFD0770c	60S ribosomal protein L15
37	PF3D7_0422500	PFD1060w	u5 small nuclear ribonucleoprotein-specific protein
38	PF3D7_0422700	PFD1070w	eukaryotic initiation factor
39	PF3D7_0503300	PFE0160c	Ser/Arg-rich splicing factor
40	PF3D7_0507100	PFE0350c	60S ribosomal protein L4
41	PF3D7_0517300	PFE0865c	putative
42	PF3D7_0520000	PFE1005w	40S ribosomal protein S9
43	PF3D7_0523000	PFE1150w	multidrug resistance protein
44	PF3D7_0529400.1	PFE1465w	conserved Plasmodium protein
45	PF3D7_0616200	PFF0785w	Ndc80 homologue
46	PF3D7_0617200	PFF0835w	conserved Plasmodium protein
47	PF3D7_0623100	PFF1110c	coronin binding protein
48	PF3D7_0905400	PFI0265c	RhopH3
49	PF3D7_0909800	PFI0475w	small nuclear ribonucleoprotein (snRNP)
50	PF3D7_0918900	PFI0925w	gamma-glutamylcysteine synthetase
51	PF3D7_1213900	PFL0675c	eukaryotic translation initiation factor 3 subunit 10
52	PF3D7_1219100	PFL0930w	clathrin heavy chain
53	PF3D7_1224000	PFL1155w	>GTP cyclohydrolase I
54	PF3D7_1224300	PFL1170w	polyadenylate-binding protein
55	PF3D7_1235900	PFL1735c	RNA-processing protein
56	PF3D7_1241200	PFL1980c	conserved Plasmodium protein
57	PF3D7_1252100	PFL2505c	crhoptry neck protein 3
58	PF3D7_1345900	MAL13P1.229	conserved Plasmodium protein
59	PF3D7_1346300	MAL13P1.233	nucleic acid binding protein
60	PF3D7_0629200	PFF1415c	DNAJ domain protein
61	PF3D7_1369700	PF13_0362	U2 small nuclear ribonucleoprotein A
62	PF3D7_0713800	PF07_0052	conserved Plasmodium protein
63	PF3D7_0935000	PFI1695c	small nuclear ribonucleoprotein (snRNP)
64	PF3D7_1437900	PF14_0359	HSP40, subfamily A
65	PF3D7_0802000	PF08_0132	glutamate dehydrogenase
66	PF3D7_1360900	MAL13P1.303	polyadenylate-binding protein
67	PF3D7_0725200	MAL7P1.139	mago nashi protein homolog
68	PF3D7_0723800	PF07_0101	conserved Plasmodium protein
69	PF3D7_0824800	PF08_0028	conserved Plasmodium membrane protein
70	PF3D7_0812700	PF08_0084	RNA-binding protein (U1 snRNP-like)
71	PF3D7_0803700	PF08_0125	tubulin gamma chain
72	PF3D7_1008800	PF10_0085	nucleolar protein NOP5
73	PF3D7_1009400	PF10_0091	zinc finger protein
74	PF3D7_1012900	PF10_0126	conserved Plasmodium protein
75	PF3D7_1102300	PF11_0035	Plasmodium exported protein
76	PF3D7_1351400	PF13_0268	60S ribosomal protein L17
77	PF3D7_1406000	PF14_0057	RNA-binding protein Alba
78	PF3D7_1415300	PF14_0151	RNA-binding protein Nova-1
79	PF3D7_1450700	PF14_0482	conserved Plasmodium protein
80	PF3D7_1461600	PF14_0587	splicing factor 3B subunit 2-like protein
81	PF3D7_1472000	PF14_0688	Pre-mRNA-splicing factor ISY1 homolog
82	PF3D7_1474500	PF14_0713	conserved Plasmodium protein
83	PF3D7_1475400	PF14_0722	cysteine repeat modular protein 4
84	PF3D7_0117800	PFB0830w	40S ribosomal protein S26e
85	PF3D7_0305300	PFC0240c	conserved Plasmodium membrane protein
86	PF3D7_0308900	PFC0375c	U2 snRNP spliceosome subunit
87	PF3D7_0310700	PFC0445w	Sybindin-like protein
88	PF3D7_0418500	PFD0895c	Bet3 transport protein
89	PF3D7_0419800	PFD0960c	60S ribosomal protein L7Ae/L30e
90	PF3D7_0922100	PFI1085w	ubiquitin-like protein
91	PF3D7_0923900	PFI1175c	>cRNA binding protein
92	PF3D7_0924700	PFI1215w	splicing factor 3A
93	PF3D7_1227600	PFL1335w	cyclin related protein
94	PF3D7_1240400	PFL1955w	erythrocyte membrane protein 1, PfEMP1
95	PF3D7_1241900	PFL2015w	similar to tetratricopeptide repeat domain 1
96	PF3D7_0706500	MAL7P1.204	conserved Plasmodium protein
97	PF3D7_1220100	PFL0970w	pre-mRNA splicing factor
98	PF3D7_1212700	PFL0625c	conserved Plasmodium protein

Supplementary Table 1. Protein list of PfCK1 interactors obtained by label-free quantitative analysis

The previous and new PlasmoDB accession numbers of proteins identified as potential PfCK1 interactors are indicated.
